# RETRACTION: Prevalence of Surgical Wound Infection and Related Factors in Patients After Long Bone Surgery: A Systematic Review and Meta‐Analysis

**DOI:** 10.1111/iwj.70296

**Published:** 2025-03-03

## Abstract

**RETRACTION:** AsadiK. , TehranyP. M. , SalariA. , VajargahP. G. , MollaeiA. , SarafiM. , AshoobiM. T. , DelshadM. S. E. , TakasiP. , FouladpourA. , KarkhahS. , FarzanR. , and ArisA. , “Prevalence of Surgical Wound Infection and Related Factors in Patients After Long Bone Surgery: A Systematic Review and Meta‐Analysis,” International Wound Journal 20, no. 10 (2023): 4349–4363, 10.1111/iwj.14300.37424390 PMC10681458

The above article, published online on 10 July 2023, in Wiley Online Library (http://onlinelibrary.wiley.com/), has been retracted by agreement between the journal Editor in Chief, Professor Keith Harding; and John Wiley & Sons Ltd. Following an investigation by the publisher, all parties have concluded that this article was accepted solely on the basis of a compromised peer review process. The editors have therefore decided to retract the article. The authors disagree with the retraction.

**RETRACTION:** K. Asadi , P. M. Tehrany , A. Salari , P. G. Vajargah , A. Mollaei , M. Sarafi , M. T. Ashoobi , M. S. E. Delshad , P. Takasi , A. Fouladpour , S. Karkhah , R. Farzan , and A. Aris , “Prevalence of Surgical Wound Infection and Related Factors in Patients After Long Bone Surgery: A Systematic Review and Meta‐Analysis,” International Wound Journal 20, no. 10 (2023): 4349–4363, 10.1111/iwj.14300.37424390 PMC10681458

The above article, published online on 10 July 2023, in Wiley Online Library (http://onlinelibrary.wiley.com/), has been retracted by agreement between the journal Editor in Chief, Professor Keith Harding; and John Wiley & Sons Ltd. Following an investigation by the publisher, all parties have concluded that this article was accepted solely on the basis of a compromised peer review process. The editors have therefore decided to retract the article. The authors disagree with the retraction.

